# “Knee-Ding” a Diagnosis: A Case of Nail Patella Syndrome

**DOI:** 10.7759/cureus.48805

**Published:** 2023-11-14

**Authors:** Sosi Dzhugarian, Sevag Hamamah, Amanda Frugoli, Angelica Shepard

**Affiliations:** 1 Graduate Medical Education, Internal Medicine, Community Memorial Hospital, Ventura, USA

**Keywords:** dysplastic patella, fong disease, hood syndrome, hereditary osteo-onychodysplasia, nail patella syndrome

## Abstract

Nail Patella Syndrome (NPS) is a rare genetic disorder with pathognomonic signs including dystrophic fingernails, iliac horns, and limb abnormalities, which commonly include hypoplastic development of the patellae, causing patients to experience patellar instability. This resulting patellar instability increases susceptibility to recurrent subluxations or dislocations in NPS patients. Since these anatomical abnormalities are present at birth or in childhood, early recognition may prevent the need for surgical intervention if appropriate preventive measures are taken. This case report describes a 54-year-old woman with a history of NPS, diagnosed later in adulthood, with a prior patellectomy at age 18 secondary to an unspecified left knee injury that occurred at age 4. A combination of radiographic and clinical findings are presented, which support the diagnosis of NPS, including dystrophic nails, left knee x-ray consistent with prior patellectomy, and right knee x-ray showing inferolateral subluxation of a hypoplastic patella. Additional signs associated with NPS are also discussed, including mood disorders, Raynaud’s, and a high hairline which may assist in early diagnosis. This case report emphasizes earlier identification of NPS by clinicians through recognition of signs and symptoms while also considering proactive measures to lessen recurrent subluxations or dislocations to preserve patellar integrity and reduce the need for surgical intervention.

## Introduction

Nail Patella Syndrome (NPS) is a rare genetic condition with an autosomal dominant inheritance pattern and a prevalence of 1/50,000 individuals [1]. NPS is also known as Fong disease as well as hereditary osteo-onychodysplasia, or hereditary onchyo-osteo-dysplasia (HOOD) syndrome [2]. The condition stems from mutations in the LMX1B gene on chromosome 9 [3,4]. There have been more than 140 described mutations including missense, frame shifting, deletion, and splicing [5]. This gene encodes a transcription factor that mediates limb dorsalization, glomerular basement membrane integrity, and dopaminergic and serotonergic neuron development [3,6-8]. Nearly all patients with NPS present with nail abnormalities, but there can be a range of deformities with the most common finding of dystrophic changes. The thumbnails are usually the most significantly affected nails, and findings decrease in severity from the lateral to medial digits [1,9]. Over 70% demonstrate pelvic girdle and limb abnormalities, commonly involving the patella and elbows [1]. There is variable renal involvement in patients with NPS, but it correlates most with mortality [2].

Unfortunately, aside from genetic counseling, there are no measures to prevent the diagnosis of NPS and no cure after diagnosis. Current treatment modalities focus on symptom management, non-surgical prevention measures to avoid limb injury, and, when indicated, surgical intervention [10,11]. Limb abnormalities of NPS commonly include patellar hypoplasia with a risk for recurrent subluxation or dislocation as well as elbow deformities that lead to an increased risk of posterior dislocation of the radial head and cubitus valgus [1]. Given the known increased risk of injury for these limb anomalies, consideration for preventative bracing during activities and some activity avoidance could be considered. Due to the rarity of this condition, activity restriction, preventative bracing would need to be individualized due to lack of specific research evidence. More importantly, these limb anomalies should not be considered in isolation, but rather as part of the NPS phenotype. 

We present a unique case of an individual with ankylosing spondylitis (AS) and NPS, with notable patellar abnormalities, who underwent a patellectomy as a young adult. In the process, we highlight important findings that are consistent with the NPS phenotype to enhance physician awareness. We then discuss how earlier recognition of disease presence could assist in initiating measures to prevent or delay the need for surgical intervention in patients with NPS limb abnormalities.

## Case presentation

A 54-year-old female with AS, NPS, and major depressive disorder (MDD) on long-term treatment with sertraline, presents with continued complaints of joint pains as well as new complaints of intermittent discoloration and coolness of her right index finger consistent with Raynaud’s. Her history is significant for an unspecified left knee injury at age 4, requiring a patellectomy at age 18. The patient has no recollection of the mechanism of injury due to her young age. However, she is certain that she was not hospitalized because of the injury and that the injury was not the result of severe trauma. She is also unaware of the exact diagnosis at the time of her patellectomy.

On review of the patient’s records, she first complained of joint pains to her primary care physician at the age of 48, which prompted an ANA screening, which yielded ANA titers of 1:160. A comprehensive ANA panel was negative for anti-dsDNA, anti-SSA/anti-SSB, and anti-Scl-70 antibodies. She did not have specific HLAB27 testing. At that time, the patient also had findings consistent with AS, including bilateral sacroiliitis, and appropriate treatment was initiated with etanercept. She also began experiencing recurrent bothersome swelling of the left knee joint, and aspiration by an orthopedist yielded 22 ml of cloudy fluid without the presence of infection or crystals. Basic laboratory evaluation demonstrated normal renal function. 

**Figure 1:**
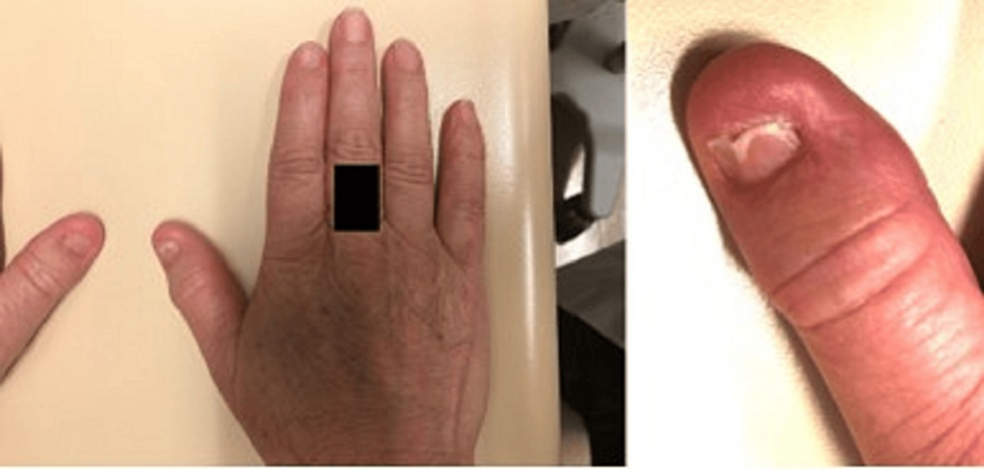
Photograph demonstrating hypoplastic nail formation on bilateral thumbs

She reports this nail formation runs in her family, including her father and son (Figure 1).

**Figure 2:**
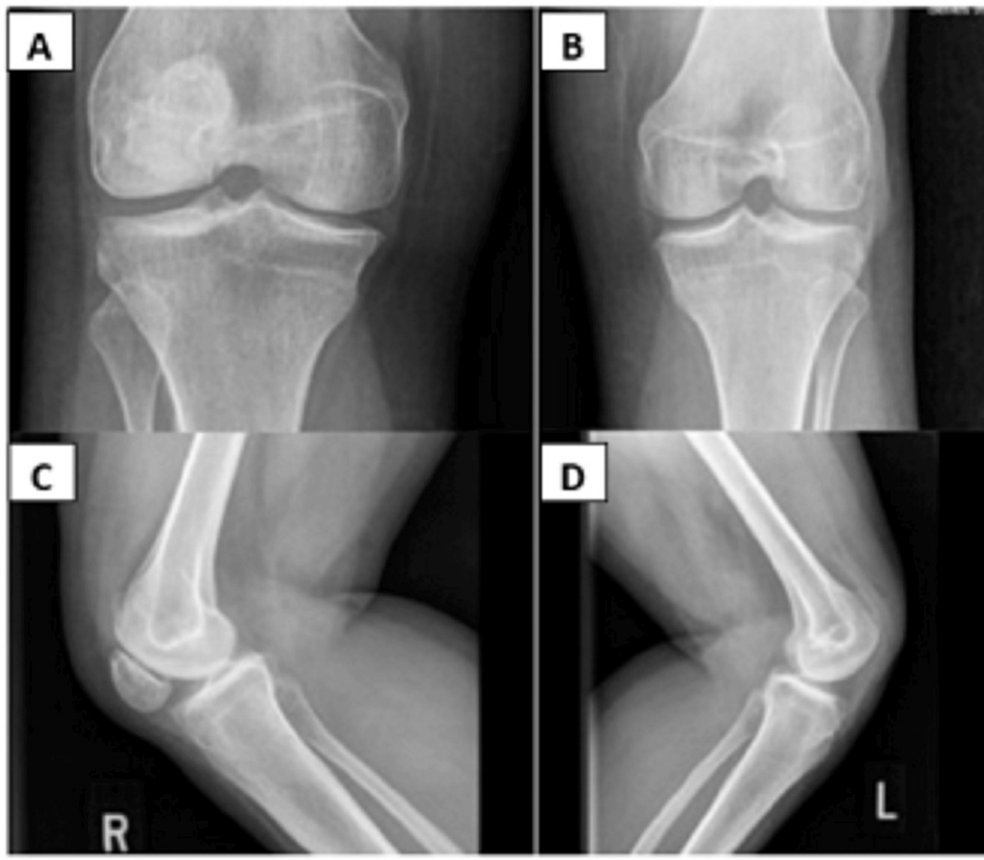
Bilateral knee radiographs with lateral and anterior-posterior (AP) views A and C demonstrate AP and lateral radiographs of the right knee with inferolateral subluxation of a hypoplastic patella; B and D demonstrate AP and lateral radiographs of the left knee consistent with prior patellectomy.

Radiographs were obtained of her knees and low back. Bilateral AP and lateral knee radiographs demonstrated inferolateral subluxation of a hypoplastic right patella and absence of the left patella consistent with prior patellectomy (Figure 2). 

**Figure 3:**
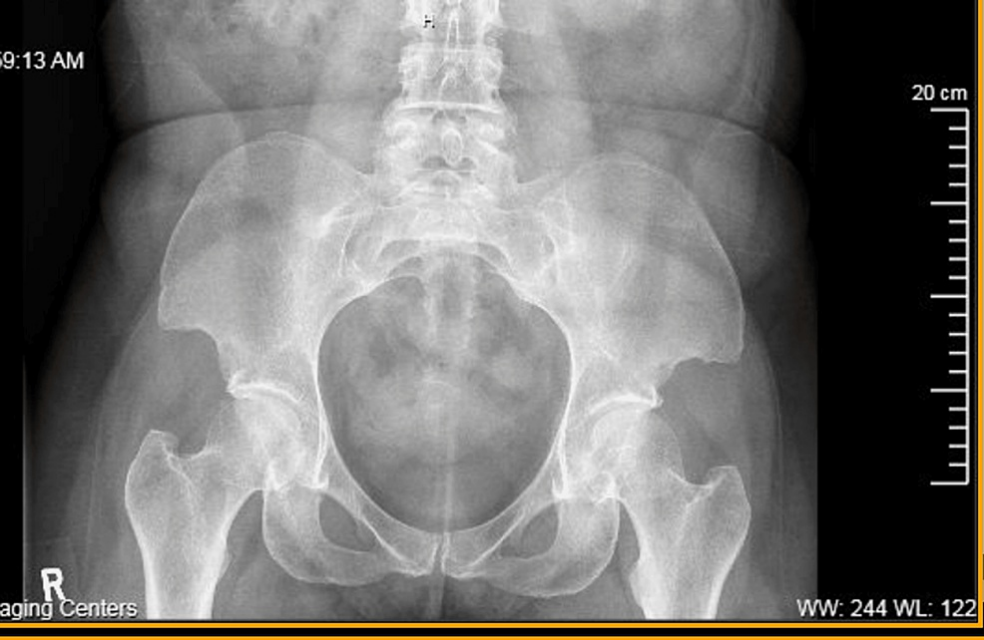
Radiograph of the pelvis. X-ray shows abnormally protuberant anterior iliac spines as well as fusion of the sacrum and ilium.

Although x-rays of the patient’s pelvis do not demonstrate pathognomonic iliac horns, they show abnormally protuberant anterior iliac spines. They demonstrate sacro-illiac fusion consistent with AS and unrelated to the presence of NPS (Figure 3).

Together, these combined findings of dystrophic nails, limb abnormalities, Raynaud’s, high hairline, history of depression, family history and negative ANA panel support the diagnosis of NPS. Our patient has not had genetic testing to determine the mutation. 

## Discussion

This case illustrates a presentation of NPS, a rare condition with wide clinical variability. Promoting increased awareness and recognition of signs and symptoms can lead to an earlier diagnosis. In our case, the patient presented with pathognomonic nail and limb abnormalities and other important associated findings consistent with the NPS phenotype including mood disorders, Raynaud’s, and a high hairline [9,11].

Subjective knee symptoms reported by NPS patients, including pain, a feeling of instability, and dislocation, are present in up to 74% of NPS cases, with hypoplasia, subluxation and dislocation of the patella as common clinical findings [9]. Our patient’s right patella was subluxated inferolaterally, and the state of her left patella prior to surgical removal is unknown. Interestingly, patellar displacement in NPS is typically superolateral [9]. Patellar instability can limit essential functions and cause debilitating pain, especially in advanced age, as the patella is important for absorbing stress from both the upper and lower legs [11-13]. Studies have shown that patellar dislocations predispose patients to earlier development of osteoarthritis and other degenerative bone changes, with an 11.9% increase in incidence at 20 years and 40.6% at 25 years [14]. The recurrence rate after a first-time patellar dislocation can be up to 44% [15,16]. Additionally, the patient’s history of left knee injury at age 4, 12 years before her patellectomy and greater than forty years prior to her official diagnosis of NPS, indicates that the patient was likely susceptible to severe patellar damage from trivial childhood activities. Given that the patella serves as an important attachment point for the quadriceps tendon, patellectomy may lead to atrophy of the quadriceps and other important leg muscles in addition to posing the general surgical risks of bleeding and infection [17]. There are reported cases of increased joint swelling after patellectomy, which can potentially explain the patient’s history of recurrent joint swelling at the left knee with at least one effusion requiring aspiration [18]. Early recognition of NPS may have prevented the need for her patellectomy at such a young age along with the subsequent symptomatic swelling and fluid aspiration in the joint. Thus, early diagnosis of patellar abnormalities may assist in utilizing safety measures to decrease the likelihood of patellar trauma and the need for surgical intervention.

Safety measures and non-operative treatment currently indicated for patellar instability include bracing, taping, immobilization, and physical therapy [19]. Leg muscle strengthening exercises, particularly for the quadriceps muscle, which consists of the rectus femoris, vastus medialis, vastus intermedius and vastus lateralis, have been shown to be effective [20]. Evidence has also supported the use of bracing or immobilization with exercise to prevent further damage or dislocation from otherwise benign movements in those with patellar instability [21]. In the case presented above, we can consider the possibility that the patient’s left patella was most likely abnormal long before her unspecified childhood injury; her dystrophic nails and history of similar nail dystrophy in her father could have alerted physicians of the presence of NPS when she was a mere toddler, instigating further work-up toward establishing a diagnosis of NPS. Assessment of her left patella with imaging could have revealed a hypoplastic and/or subluxed left patella, similar to her right, long before her reported injury. Given that she likely had patellar instability at baseline, an earlier diagnosis and implementation of preventative measures may have allowed for conservative management and prevented the need for patellectomy altogether.

Although there are studies on prophylactic knee bracing, they focus on the setting of preventing patellar injuries in athletes or post-surgical patients and have so far been inconclusive. A future study to consider is analysis of the use of prophylactic hinged knee braces in patients diagnosed with NPS, although such a study may be difficult to conduct given the rarity of the disease and the accompanying variability in patellar findings.

Another study to consider involves exploring whether certain activities should be avoided in individuals with NPS; it can be further subdivided into studies based on age or activity. Whether as children or adults, perhaps certain sports should be avoided, such as soccer which involves knee pivoting, or volleyball which can consist of repetitive knee flexion. As teenagers and adults, perhaps certain exercises should be avoided, such as squatting with heavy weights. There should be analysis of whether certain occupations which involve excessive kneeling or stair climbing can increase or hasten the need for patellectomy, such as agriculture, construction, or firefighting. Such studies can help establish guidelines for physicians and patients regarding implementation of safety measures in lieu of or in addition to orthotics.

Our patient had full range of motion of the elbow, without abnormalities on inspection, but NPS can effect this joint as well. The elbow findings may not necessarily be symmetrical and may include limited range of motion with pronation, supination and extension. On inspection cubitus valgus may be present as well as pterygia at the antecubital region [9]. Notably, imaging may demonstrate dysplasia of the radial head, along with hypoplasia of the lateral epicondyle and capitulum. These anatomical deviations can increase the risk of dislocation of the radial head [9]. Similar to the consideration of utilizing knee braces as preventative measures, perhaps elbow bracing in select patients could be beneficial. 

It is important to note that the effects of NPS are not limited to the integumentary and musculoskeletal systems. The genetic mutations can affect the renal glomeruli and lead to abnormalities in the glomerular basement membrane [6]. Therefore, regular monitoring of NPS patients is required, especially since this portends the greatest influence of mortality [22]. Fortunately, our patient’s renal function was normal as evidenced by laboratory assessment.

Studies have also shown that individuals diagnosed with NPS are at increased risk of developing mood disorders as compared to the general population, with one study of 50 NPS patients showing a 22% increase in Conners’ Adult ADHD Scales Scores and 40% increase in Beck Depression Inventory-II scores [12]. These findings can be explained by gene knockout studies that have shown that the loss of function mutation in the LMX1b gene leads to abnormal development of the serotonergic and mesencephalic dopaminergic neurons [23]. More specifically, axonal projections of 5-HT neurons to the forebrain and spinal cord in LMX1b deficient individuals fail to generate [24]. As such, successive morphological events that mediate post-natal serotonergic neuronal development are impaired and neurotransmission and neuromodulation of serotonergic signaling can be affected, increasing risk for comorbid depression. In the case of our patient, her comorbid depression was well-controlled by sertraline, a serotonin-selective reuptake inhibitor that can increase bioavailability of serotonin, by increasing the presence of serotonin in the synaptic cleft.

Furthermore, vasomotor abnormalities, such as Raynaud’s phenomenon, described by the patient as intermittent discoloration and coolness of her right index finger, are another common finding associated with NPS [1]. In their study, Sweeney et al. showed that 12/22 of NPS patients reported symptoms of poor peripheral circulation such as numbness, tingling, burning or discoloration with some formally diagnosed with Raynaud’s phenomenon [9]. This can be potentially explained by the role of LMX1b in limb dorsalization, where deficiency of the gene can lead to aberrant migration of neuronal cells to digital cutaneous nerves [9]. Peripheral nerve endings are shown to sense environmental modifications, such as temperature changes, controlling vascular tone in the digital arteries and cutaneous arterioles [25]. Therefore, the reduction in nerve ending density in NPS patients can cause blood-flow restrictions, manifesting as vasomotor symptoms in distal extremities.

Due to its rarity among the general population, NPS may go undiagnosed for years, through childhood, adolescence, and well into adulthood, as was the case of our patient. Recognizing the signs and symptoms of the disease and initiating the proper work-up can allow clinicians to decrease or delay the consequences of the progression of the condition’s effect on different parts of the body. The classic case presented above consisting of nail, iliac, and patellar findings, pathognomonic of NPS, demonstrates the need to increase physician awareness of this rare condition which requires long-term surveillance. Further research is required to develop evidence-based guidelines on disease surveillance. Some experts recommend annual screening for renal disease with systolic blood pressure measurements, serum creatinine and urine analysis. Screening for glaucoma on a regular basis is also recommended. All patients with NPS should be offered genetic counseling [1,2,9]. 

Since the nail findings of NPS are present in almost all affected individuals, they should be utilized to alert physicians of disease presence. Diagnosis is key to addressing subsequent long-term care concerns with annual monitoring of renal function. This also includes initiation of safety measures in those found to have concurrent patellar abnormalities in order to decrease the likelihood of patellar injury and prevent the need for surgical intervention.

## Conclusions

This case report emphasizes earlier identification of NPS by clinicians through recognition of signs and symptoms while also considering proactive measures to lessen recurrent subluxations or dislocations to preserve patellar integrity and reduce the need for surgical intervention. Physicians should also recognize that the effects of NPS are not limited to the integumentary and musculoskeletal systems and patients should have ongoing surveillance for other related co-morbidities including depression and renal manifestations. 
